# *Lactobacillus salivarius* Subspecies *salicinius* SA-03 is a New Probiotic Capable of Enhancing Exercise Performance and Decreasing Fatigue

**DOI:** 10.3390/microorganisms8040545

**Published:** 2020-04-09

**Authors:** Mon-Chien Lee, Yi-Ju Hsu, Hsieh-Hsun Ho, Shih-Hung Hsieh, Yi-Wei Kuo, Hsin-Ching Sung, Chi-Chang Huang

**Affiliations:** 1 Graduate Institute of Sports Science, National Taiwan Sport University, No. 250, Wenhua 1st Rd., Guishan District, Taoyuan City 33301, Taiwan; 1061304@ntsu.edu.tw (M.-C.L.); ruby780202@ntsu.edu.tw (Y.-J.H.); 2 Glac Biotech Co. Ltd., Tainan City 74442, Taiwan; sam.ho@glact.com.tw (H.-H.H.); shih-hung.hsieh@glact.com.tw (S.-H.H.); vic.kuo@glact.com.tw (Y.-W.K.); 3 Aesthetic Medical Center, Department of Dermatology, Chang Gung Memorial Hospital, Taoyuan 33301, Taiwan; 4 Department of Anatomy, College of Medicine, Chang Gung University, No. 259, Wenhua 1st Rd., Guishan Township, Taoyuan City, Taoyuan 33301, Taiwan

**Keywords:** probiotic, SA-03, *Lactobacillus*, nutritional supplement, exercise performance, weightlifting

## Abstract

Probiotics are increasingly being used as a nutritional supplement by athletes to improve exercise performance and reduce post-exercise fatigue. *Lactobacillus salivarius* is a natural flora in the gastrointestinal tract of humans and animals. *Lactobacillus salivarius* subspecies *salicinius* (SA-03) is an isolate from the 2008 Olympic women’s 48 kg weightlifting gold medalist’s gut microbiota. In this study, we investigated its beneficial effects on physical fitness. Male ICR mice were divided into four groups (*n* = 10 per group) and orally administered with SA-03 for 4 weeks at 0, 2.05 × 10^9^, 4.10 × 10^9^, or 1.03 × 10^10^ CFU/kg/day. Results showed that 4 weeks of SA-03 supplementation significantly improved muscle strength and endurance performance, increased hepatic and muscular glycogen storage, and decreased lactate, blood urea nitrogen (BUN), ammonia, and creatine kinase (CK) levels after exercise. These observations suggest that SA-03 could be used as a nutritional supplement to enhance exercise performance and reduce.

## 1. Introduction

Non-pathological / physiological fatigue can be further characterized as being caused by central and peripheral mechanisms, and both of these mechanisms play important roles in the physiological effects of exercise process, type, intensity, and duration [1,2]. During prolonged endurance exercise, the energy resources required by muscles are insufficient to maintain and produce the same level of strength, causing fatigue reactions and decrease exercise performance [3]. As people become more interested in the gut microbiota a relationship with exercise performance was found. Gut microbiota can ferment dietary fiber to short-chain fatty acids (SCFA) as an energy source for liver and muscle cells and improve endurance performance through stable blood glucose regulation. In addition, SCFA seems to be able to reduce the permeability of colonic mucosa by regulating the function and metastasis of neutrophils, inhibiting inflammatory cytokines and reducing the redox response, which is one of the possible factors to reduce fatigue [4,5].

In recent years, sports nutrition supplements are considered as necessary for increasing exercise performance or reducing post-exercise fatigue and have played an important role in scientific exercise training. In addition to traditional herbs, proteins and creatine, probiotics have also received increasing attention as dietary supplements. According to the World Health Organization, Food and Agriculture Organization of the United Nations (WHO/FAO) definition, probiotics are live microorganisms, not causing any adverse effects on the organism and providing health benefits when administered in appropriate amounts [6]. Previous studies have shown that probiotics have anti-obesity effects [7], can lower cholesterol levels [8], anti-inflammation [9], anti-bacterial [10], anti-oxidation [11], anti-proliferative and anti-carcinogenic activities [12]. They have also been shown to enhance carbohydrate metabolism to produce SCFA [13] such as butyrate, which is then converted to acetyl-CoA for production of adenosine triphosphate (ATP) to provide energy [14]. Therefore, past research has shown that, probiotic supplements can effectively improve exercise performance and reduce fatigue biochemical values after exercise [15,16].

The name *Lactobacillus salivarius* derives from the characteristic “saliva” of the oral cavity, from which it was isolated for the first time [17]. Research findings in recent years have shown that *L. salivarius* is commonly found in the gastrointestinal tract of humans and animals. The genomic structure of *L. salivarius* was first derived from the UCC118 chromosome. The strain′s genome consists of a 1.83 Mb chromosome, a large 242 kb plasmid, and two smaller plasmids [18]. Its genomic diversity has recently been well evaluated and has been studied as a candidate probiotic. [19]. Preliminary studies have described the species’ immunomodulatory properties in cell lines, mice, rats, and humans to eliminate diseases in the body and promote host health. The ability of *L. salivarius* to suppress the resistance of pathogens and hosts to antibacterial defense demonstrates the adaptability of the species′ hormone niche [20]. Previous studies have shown that *L. salivarius* supplementation reduces exercise-induced gastrointestinal permeability and remodels the gut microbiome in healthy humans [21], however, the evolutionary species and subspecies of different strains have different functional characteristics. Emerging research on the probiotic potential of this species remains to be discussed in detail.

*Lactobacillus salivarius* subsp. * salicinius* (SA-03), a human-origin probiotic was isolated from a female weightlifter’s gut microbiota. In this study, we evaluated the function and efficacy of SA-03 supplements through exercise testing and analysis of fatigue-related biochemical indicators, and confirmed that it would not cause adverse damage in mice.

## 2. Materials and Methods

### 2.1. Lactobacillus salivarius subsp. salicinius (SA-03) Preparation

*Lactobacillus salivarius* SA-03 was isolated from the 2008 Olympic women’s 48 kg weightlifting gold medalist’s gut microbiota. The isolate was confirmed to be *Lactobacillus salivarius* by the Food Industry Research and Development Institute (Hsinchu, Taiwan). The dry product of SA-03 was prepared and provided by Glac Biotech Co., Ltd. (Tainan City, Taiwan). Viable cell counts of SA-03 were 1.07 × 10^11^ CFU/g. The powder was suspended in phosphate buffered saline (PBS, pH 7.2) before consumption.

### 2.2. Animals and Experimental Design

Pathogen-free male 6-week-old ICR mice were purchased from BioLASCO (Yi-Lan, Taiwan). All animal experiments were approved by the Institutional Animal Care and Use Committee (IACUC) of National Taiwan Sport University. Mice were maintained at a 12-h light/dark cycle at room temperature (23 ± 2 °C) and 50–60% humidity and provided with reverse osmosis (R.O) water and standard chow diet (No. 5001; PMI Nutrition International, Brentwood, MO, USA) ad libitum.

As probiotics are being used in humans at a daily dose of 1 × 10^10^ live bacteria [22], the dose given to mice was converted from the human equivalent dose (HED) based on the surface area of human body. Accordingly, mice were divided into four treatment groups (a total of three cages per group, each containing 3-4 mice per cage, 10 mice total per group): (1) vehicle group (0 CFU/kg); (2) SA-03-1X group (2.05 × 10^9^ CFU/kg); (3) SA-03-2X group (4.10 × 10^9^ CFU/kg), and (4) SA-03-5X group (1.03 × 10^10^ CFU/kg). All mice were administered by gavage the same volume of SA-03 suspension or PBS daily for 4 weeks. 

### 2.3. Swimming Exercise Endurance Test

The swim-to-exhaustion exercise test was performed as previously described [23]. A weight equivalent to 5% of body weight (BW) was loaded on the tail of the test mouse. The test mouse was then forced to swim until loss of coordinated movements or failure to return to the surface within 7 s [24], and the duration of such swimming was recorded.

### 2.4. Forelimb Grip Strength

A low-force testing system (Model-RX-5, Aikoh Engineering, Nagoya, Japan) with a tension rod (diameter 2 mm, length 7.5 cm) and force sensor was used to measure the grip strength of mice as described previously [25]. 

### 2.5. Determination of Fatigue-Associated Biochemical Variables

As previous reports [26], post-exercise assessment of the effects of supplementation SA-03 on fatigue-related biochemical indicators, and accurate display and assessment of physiological status. Fatigue-related variables were measured under fasting conditions to reflect actual physiological fitness. under exercise. intervention. Blood samples were collected after 10 min of swimming and 20 min of rest. The samples were centrifuged at 1500 x g for 15 min at 4 ° C, and serum was collected to analyzed. The Serum levels of lactic acid, ammonia (NH_3_), and glucose levels were measured by with an automatic analyzer (model 7060, Hitachi, Tokyo, Japan). Other variables, such as blood urine nitrogen (BUN) and creatine kinase (CK), were evaluated immediately after 90 min of extended exercise and 60 min of rest.

### 2.6. Resting Biochemical Profiles at the End of the Study

One hour after the last swimming endurance test, all mice were euthanized by 95% CO2 asphyxiation, and blood was obtained by cardiac puncture at the end of the study. Serum was collected after centrifugation and biochemical indexes assessed by a Hitachi 7060 autoanalyzer. Levels of aspartate aminotransferase (AST), alanine transaminase (ALT), albumin (ALB), total protein (TP), BUN, creatinine (CREA), uric acid (UA), total cholesterol (TC), triglycerides (TG), creatine kinase (CK), and glucose (GLU) were measured with the aforementioned autoanalyzer.

### 2.7. Body Composition, Glycogen Content, and Histopathology

After being euthanized, the liver, kidneys, heart, lungs, muscles, epididymal fat pad (EFP), and brown adipose tissue (BAT) of mice were excised, weighed, and processed for histological examinations. Tissue sections were stained with hematoxylin and eosin (H & E) and examined by a clinical pathologist. Muscle and liver tissues were also processed for determination of glycogen content. 

### 2.8. Bacterial DNA Extraction and 16S rRNA Sequencing

According to the method previously used in our laboratory, immediately after euthanizing the mice, the collected samples were stored at -80 ° C for DNA extraction. Detailed procedures for sample extraction, preparation and analysis have been previously described [16].

### 2.9. Statistical Analysis

All statistical analyses were performed with SAS 9.4 (SAS Institute, Cary, NC, USA). One-way analysis of variance (ANOVA) was used to determine significant difference among groups. The Cochran–Armitage test was used for dose-effect trend analyses. Data are expressed as mean ± SD. *p* < 0.05 was considered as significant.

## 3. Results

### 3.1. Effect of SA-03 Supplementation on Body Weight, Body Composition, and Food and Water Intake

**Figure 1:**
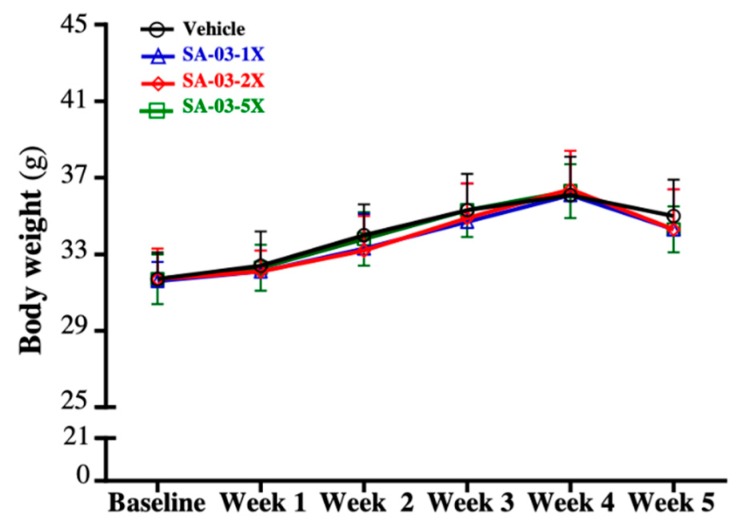
Effect of SA-03 supplementation on body weight of mice. Data are expressed as mean ± SD for *n* = 10 mice per group.

There was no significant difference in average body weight of mice among the 4 different dosing groups at different time points during the four weeks of SA-03 supplementation. (Figure 1). There was also no significant difference in food and water intake and various parameters among the four groups (Table 1).

### 3.2. Effect of SA-03 Supplementation on Endurance Capacity

**Figure 2:**
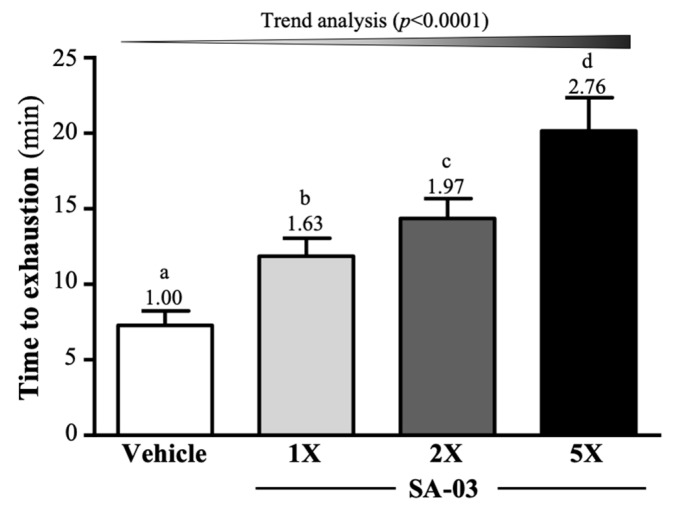
Effect of four weeks of SA-03 supplementation on exhaustive swim time. Data are expressed as mean ± SD (*n* = 10 mice per group). Different superscript letters (a, b, c, d) indicate significant difference (*p* < 0.05).

The average exhaustive swim time of mice in vehicle, SA-03-1X, SA-03-2X, and SA-03-5X groups was 7.29 ± 0.95, 11.86 ± 1.19, 14.37 ± 1.30, and 20.15 ± 2.21 min, respectively (Figure 2). Compared to the vehicle group, the average exhaustive swim time of SA-03-1X, SA-03-2X, and SA-03-5X groups was increased by 1.63-fold (*p* < 0.0001), 1.97-fold (*p* < 0.0001), and 2.76-fold (*p* < 0.0001), respectively after four weeks of SA-03 supplementation. Results of trend analyses showed that the effect of SA-03 supplementation on maximum swim time was dose dependent (*p* < 0.0001).

### 3.3. Effect of SA-03 Supplementation on Grip Strength

**Figure 3:**
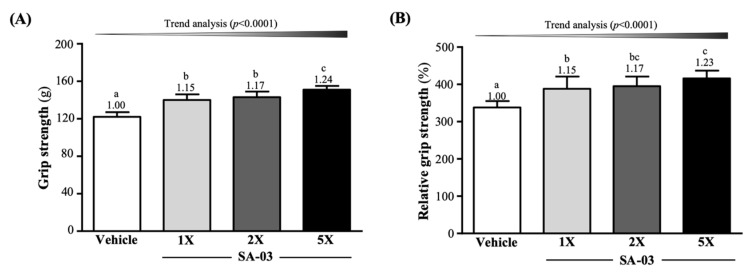
Effect of four weeks of SA-03 supplementation on (**A**) absolute forelimb grip strength and (**B**) forelimb grip strength (%) relative to body weight. Data are expressed as mean ± SD (*n* = 10 mice per group). Different superscript letters (a, b, c) indicate significant difference (*p* < 0.05).

The mean forelimb grip strengths of mice in vehicle, SA-03-1X, SA-03-2X, and SA-03-5X groups were 122 ± 5, 140 ± 6, 143 ± 6, and 151 ± 4 g (Figure 3A), respectively after 4 weeks of SA-03 supplementation. This represented a 1.15-fold (*p* < 0.0001), 1.17-fold (*p* < 0.0001), and 1.23-fold (*p* < 0.0001) increase in grip strength of mice in the three treatment groups, respectively, compared to mice in the vehicle group. Relative grip strength (%), normalized to body weight, was also significantly higher in groups with SA-03 supplementation (Figure 3B). The effect of SA-03 supplementation on both absolute and relative grip strength was dose dependent (trend analysis, *p* < 0.0001).

### 3.4. Effect of SA-03 Supplementation on Serum Lactate Levels after the 10-Min Swim Test

After 4 weeks of SA-03 supplementation, mice were subjected to the 10-min swimming test and assessed for serum lactate levels at three time points: pre-exercise, post-exercise, and 20 min after resting (Table 2). Before swimming, there was no significant difference in the levels of serum lactate among the four groups. After 10 min of swimming, serum lactate levels of mice in vehicle, SA-03-1X, SA-03-2X, and SA-03-5X groups were 8.73 ± 0.65, 7.53 ± 0.90, 6.65 ± 0.85, and 4.53 ± 0.89 mmol/L, respectively. This result indicated a decrease of 13.80% (*p* = 0.0012), 23.89% (*p* < 0.0001), and 33.77% (*p* < 0.0001) in serum lactate levels in SA-03-1X, SA-03-2X, and SA-03-5X groups, respectively, compared to the vehicle group. Based on serum lactic acid concentration before and after 10 min of swimming, the lactate production rates were determined to be 2.64 ± 0.47, 2.30 ± 0.41, 2.13 ± 0.57 and 1.68 ± 0.30, respectively in vehicle, SA-03-1X, SA-03-2X, and SA-03-5X groups. Compared to the vehicle group, the lactate production rate of mice in the SA-03-2X group was decreased by 19.17% (*p* = 0.0156) and that of mice in the SA-03-5X group was decreased by 36.47% (*p* < 0.0001) after four weeks of SA-03 supplementation.

After 20 min of resting following the swimming test, serum lactate levels of mice in vehicle, SA-03-1X, SA-03-2X, and SA-03-5X groups were 6.84 ± 0.48, 5.85 ± 1.08, 5.40 ± 0.71, and 4.53 ± 0.89 mmol/L, respectively. The levels of mice in the three treatment groups were 14.56% (*p* = 0.0099), 21.16% (*p* = 0.0003), and 33.76% (*p* < 0.0001), respectively, lower than those of mice in the vehicle group. The effect of SA-03 supplementation on serum lactate levels was also dose dependent.

### 3.5. Effect of SA-03 Supplementation on Serum Glucose and Ammonia Levels after the 10 Min Swim Test

**Figure 4:**
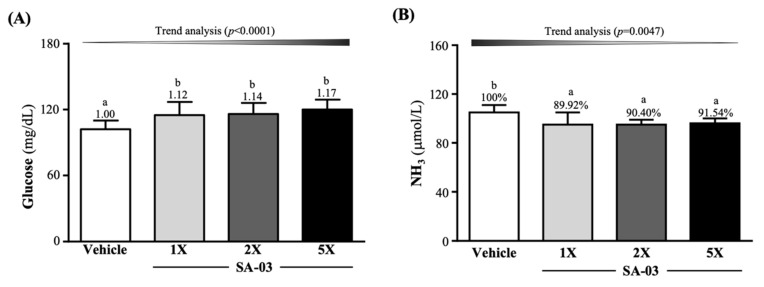
Effect of 4 weeks of SA-03 supplementation on serum (**A**) glucose and (**B**) ammonia (NH_3_) levels after 10 min of swimming. Data are expressed as mean ± SD (*n* = 10 mice per group). Different superscript letters (a, b) indicate significant difference (*p* < 0.05).

As shown in Figure 4A, serum glucose levels of mice after 10 min of swimming in vehicle, SA-03-1X, SA-03-2X, and SA-03-5X groups were 102 ± 8, 115 ± 12, 116 ± 10, and 120 ± 9 (mg/dL). The levels of the three treatment groups were 1.12-fold (*p* = 0.0098), 1.14-fold (*p* = 0.0035), and 1.17-fold (*p* = 0.0004) that of the vehicle group, respectively. Serum ammonia levels of mice in vehicle, SA-03-1X, SA-03-2X, and SA-03-5X groups were 105 ± 6, 95 ± 10, 95 ± 4, and 96 ± 4 (µmol/L), respectively, and the levels of the three treatment groups were 10.08% (*p* = 0.0010), 9.60% (*p* = 0.0015), and 8.46% (*p* = 0.0046) lower than that of the vehicle group, respectively.

### 3.6. Effect of SA-03 Supplementation on Serum BUN and CK Levels after 90 Min Swimming and 60 Min Rest

**Figure 5:**
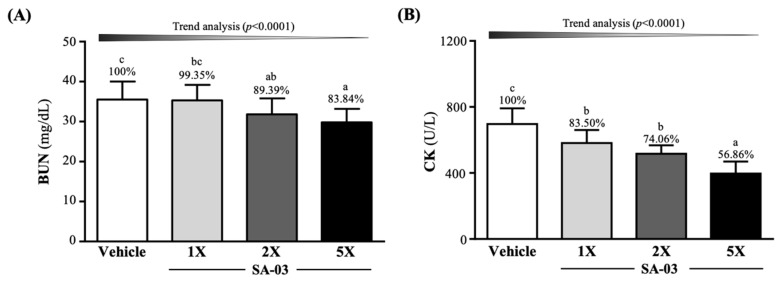
Effect of 4 weeks of SA-03 supplementation on serum (**A**) BUN and (**B**) CK levels after 90-min swimming exercise and 60-min rest. Data are expressed as mean ± SD (*n* = 10 mice per group). Different superscript letters (a, b, c) indicate significant difference at *p* < 0.05.

To study the anti-fatigue effect of SA-03, serum BUN levels were measured 60 min after the 90-min swimming test (Figure 5A). Serum BUN levels were found to be 35.5 ± 4.5, 35.3 ± 3.9, 31.8 ± 4.0, and 29.8 ± 3.4 mg/dL in mice in vehicle, SA-03-1X, SA-03-2X, and SA-03-5X groups, respectively. This result indicated that exercise-induced BUN was decreased by 10.61% (*p* = 0.0416) in mice in the SA-03-2X group and 16.16% (*p* = 0.0027) in those in the SA-03-5X groups.

CK levels, an exercise injury index, were found to be decreased by 16.50% (*p* = 0.0019), 25.94% (*p* < 0.0001), and 43.14% (*p* < 0.0001), respectively, in SA-03-1X, SA-03-2X, and SA-03-5X groups compared to the vehicle control group.

### 3.7. Effect of SA-03 Supplementation on Liver and Muscle Glycogen Levels

**Figure 6:**
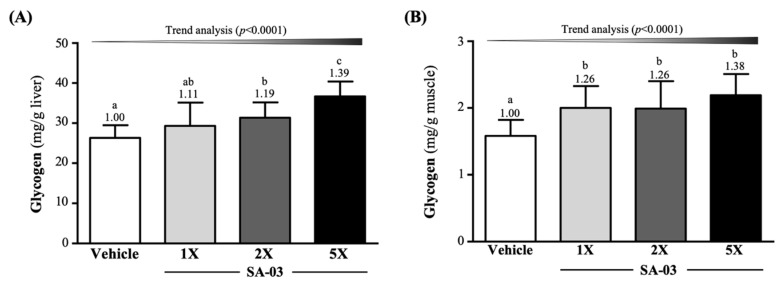
Effect of 4 weeks of SA-03 supplementation on (**A**) hepatic and (**B**) muscle glycogen levels. Data are expressed as mean ± SD for *n* = 10 mice per group. Values with different superscript letters (a, b, c) are significantly different at *p* < 0.05.

Liver glycogen levels of mice in vehicle, SA-03-1X, SA-03-2X, and SA-03-5X groups were 26.32 ± 3.14, 29.33 ± 5.83, 31.35 ± 3.82, and 36.69 ± 3.69 mg/g of liver, respectively and were elevated by 1.19-fold (*p* = 0.0120) in the SA-03-2X group and 1.39-fold (*p* < 0.0001) in the SA-03-5X group compared to the vehicle group (Figure 6A). Muscle glycogen levels in vehicle, SA-03-1X, SA-03-2X, and SA-03-5X groups were 1.58 ± 0.24, 2.00 ± 0.33, 1.99 ± 0.41, 2.19 ± 0.32 mg/g of muscle, representing an increase of 1.26-fold (*p* = 0.0084), 1.26-fold (*p* < 0.0098), and 1.38-fold (*p* = 0.0002) in the three treatment groups, respectively (Figure 6B). The effect of SA-03 supplementation on hepatic and muscular glycogen content was also dose dependent (*p* < 0.0001).

### 3.8. Effect of SA-03 Supplementation on Biochemical Profiles

The effect of SA-03 supplementation on biochemical parameters were also evaluated (Table 3). The levels of liver damage markers (AST and ALT) and mean levels of albumin, TC, TG, CK, BUN, creatinine, UA, TP, and glucose were found to be similar among the groups (*p* > 0.05). This observation suggests that SA-03 has no adverse effects on health.

### 3.9. Effect of SA-03 Supplementation on the Gut Microbiota

**Figure 7:**
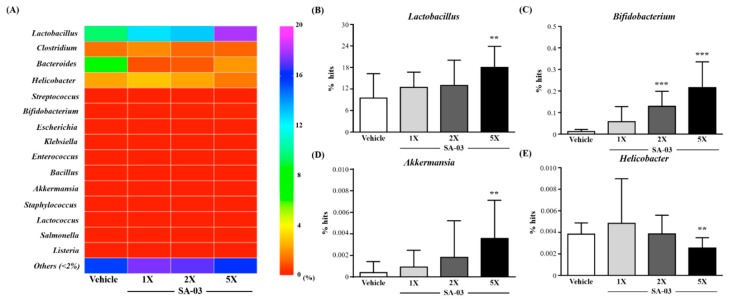
Effect of 4 weeks of SA-03 supplementation on (**A**) *Genus* heatmap of gut microbiota, (**B**) *Lactobacillus* % hit, (**C**) *Bifidobacterium* % hit, (**D**) *Akkermania* % hit *and* (**E**) *Helicobacter* % hit. Data are expressed as mean ± SD for *n* = 9 mice per group. Values with different superscript letters are significantly different at *, *p* < 0.05; **, *p* < 0.05; ***, *p* < 0.05 by *t*-test.

We analyzed the gut microbiota composition using the 16S rRNA *Genes* in the vehicle or SA-03- treated mice and observed dramatic changes in the microbial ecology when treated with SA-03 at the end of experiment. As shown in Figure 7A, the number of *Lactobacillus*, *Bifidobacterium*, *Enterococcus*, *Akkermania*, and *Lactococcus* in SA-03 supplementation were all richer than vehicle at the level of microbiota, especially in the *Lactobacillus* (Figure 7B), *Bifidobacterium* (Figure 7C) and *Akkermania* (Figure 7D), the ratio of SA-03-2X and 5X were significantly richer (*p* < 0.05). In addition, among the human harmful gut microbiota, the Clostridium, *Helicobacter*, *Escherichia*, and *Listeria* was lower in the SA-03-5X group than vehicle group, especially *Helicobacter* (Figure 7E) was significantly lower (*p* < 0.05).

**Figure 8:**
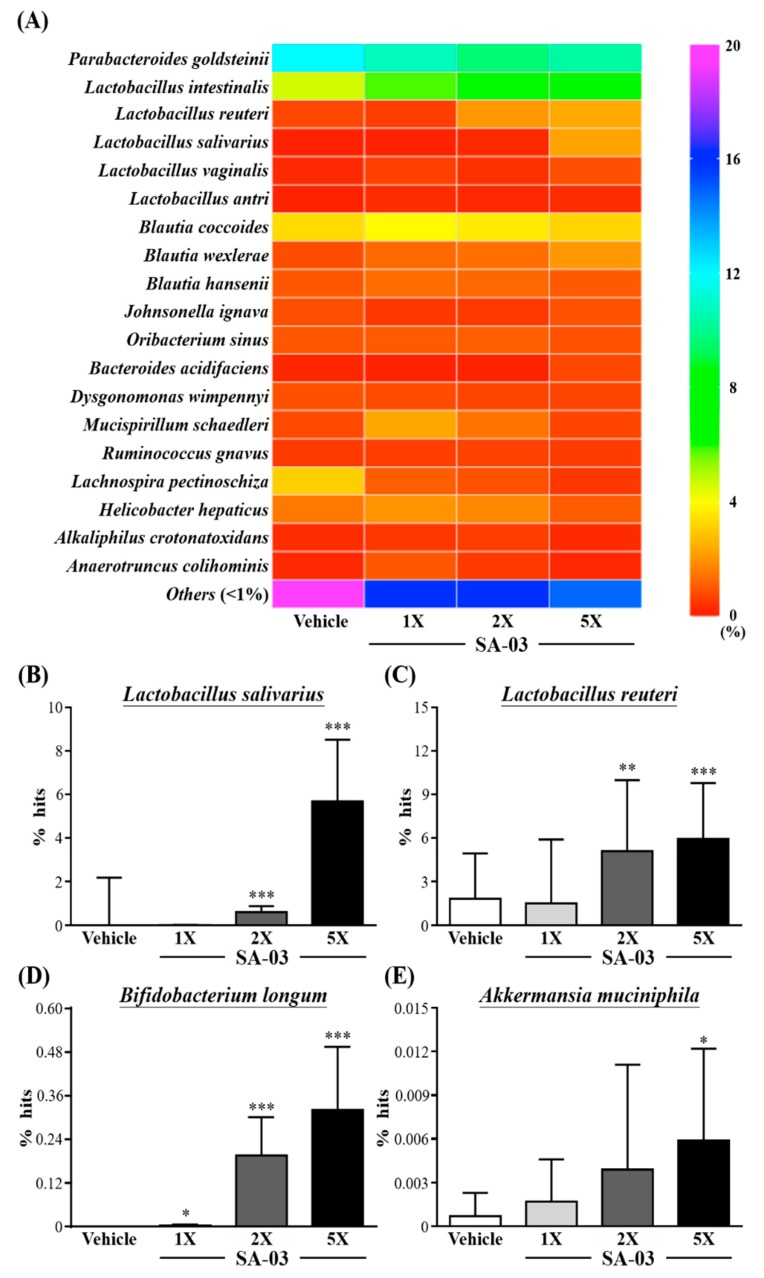
Effect of 4 weeks of SA-03 supplementation on (**A**) *Species* heatmap of gut microbiota, (**B**) *Lactobacillus salivarius* % hit, (**C**) *Lactobacillus reuteri* % hit, (**D**) *Bifidobacterium longum* % hit and (**E**) *Akkermansia muciniphila* % hit. Data are expressed as mean ± SD for *n* = 9 mice per group. Values with different superscript letters are significantly different at *, *p* < 0.05; **, *p* < 0.05; ***, *p* < 0.05 by *t*-test.

In *Species* (Figure 8A)*,* the number of *Lactobacillu* in SA-03 supplementation of mice were total increase, among them, *Lactobacillus salivarius*, *Lactobacillus reuteri*, *Lactobacillus vaginalis*, and *Lactobacillus antri* were more abundant than the vehicle group. In comparison, the percentage changes of *Lactobacillus* species in SA-03-1X, SA-03-2X and SA-03-5X were 4.23%, 9.07%, and 16.55%, respectively.

Compared with vehicle group, the *Lactobacillus salivarius* (Figure 8B) in Sa-03-1X, SA-03-2X and SA-03-5X were significantly increased and the percentage were changes by 0.01%, 0.61% and 5.68% (*p* < 0.001), respectively. The abundance of *Lactobacillus reuteri* (Figure 8C) also significantly increased in SA-03-2x and SA-03-5x (*p* < 0.01) 3.27% and 4.11% percentage changes, respectively. In particular, in addition to the significantly increased in the richness of *lactobacillus* by SA-03 supplementation, *Bifidobacterium longum* (Figure 8D) and *Akkermansia muciniphila* (Figure 8E) percentage of gut microbiota were significantly richer than vehicle group (*p* < 0.05). In addition, among the human harmful gut microbiota, the *Helicobacter hepaticus* was significantly lower in the SA-03-5X group than vehicle group, from 3.74% to 2.64% (*p* < 0.05).

### 3.10. Effect of SA-03 Supplementation on Tissue Histology

**Figure 9:**
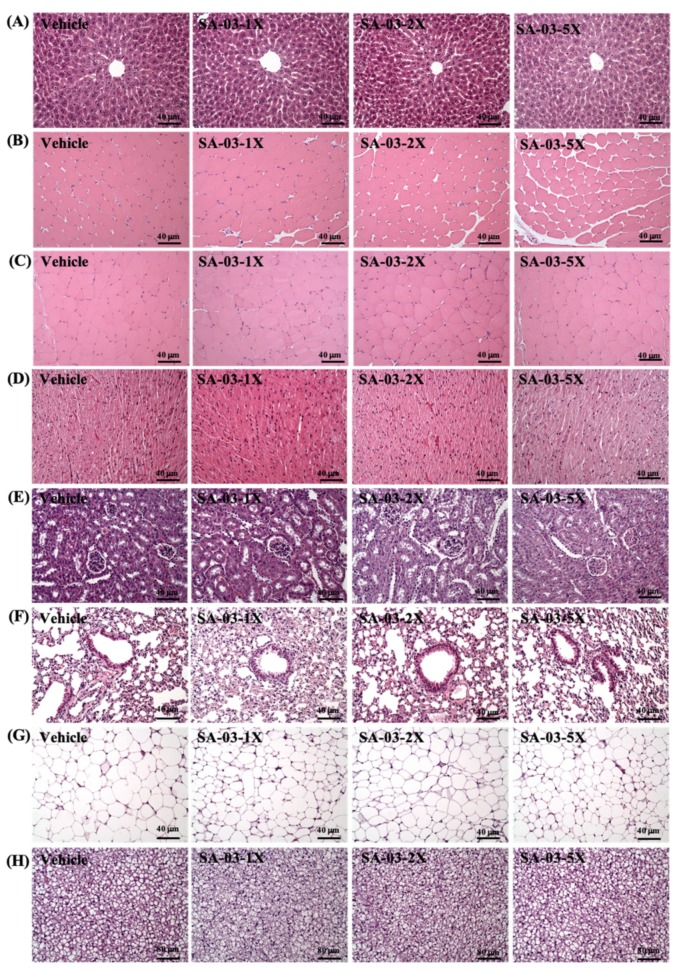
Effect of SA-03 supplementation on (**A**) liver, (**B**) muscle, (**C**) quadricep muscles, (**D**) heart, (**E**) kidney, (**F**) lung, (**G**) adipocyte tissue, and (**H**) BAT tissue in mice. (H&E stain, magnification: 200×; bar, 40 μm; BAT magnification: 100×; bar, 80 μm).

At the end of the study, histological examinations of liver, muscle, heart, kidney, lung, EFP and BAT of mice were performed, and no abnormalities were observed among all groups (Figure 9). There was also no difference in the morphology of adipose tissue and fat cell size among the groups. These results indicate that SA-03 does not have adverse effects on organs and tissues at the doses tested in this study.

## 4. Discussion

Although different strains have different efficacy characteristics, research specifically designed to investigate the effect of probiotic supplementation on performance is still uncommon. Recent studies have shown that probiotic supplements can improve athletic performance in various ways through athletes and individuals who exercise with discrete probiotic strains and pointed out that they could effectively improve exercise performance and reduce fatigue indicators [27]. However, the probiotic effect may relate to the strain, dose, period consumption or even the form of administration (capsules, sachets or fermented milk) [5]. Therefore, we used the recommended human dose of 1x10^10^ CFU/day as 1 times dose [22] and following required by the anti-fatigue health food regulations established by the Taiwan Ministry of Health and Welfare to design the 1, 2, and 5 times doses used in this study. Four weeks of *L. salivarius* (SA-03) supplementation was found to significantly improve muscle strength and weight-loading swimming endurance, reduce serum levels of fatigue indicators (lactate, BUN, ammonia, and CK), and increase liver and muscle glycogen content. SA-03 was also found to have no effects on organs and tissues.

There is a close relationship between changes in intestinal microorganisms and athletic performance. Several studies have found that that athletes have increased intestinal microbial diversity compared to non-athletes [28,29,30]. Moreover, another study has also been observed that exercise is directly proportional to the abundance of pathways, which in turn is related to the increased branched chain amino acid (BCAA) pathway [28], which is important for muscle recovery and increase in *Methanobrevibacter smithii*, a microorganism that uses H_2_ in the colon to produce SCFA and ATP [29]. Therefore, it seems reasonable that probiotics can improve athletic performance through the action of probiotics, because athletes can delay fatigue by producing SCFA. In this study, we found that four weeks of SA-03 supplementation significantly decreased one of the fatigue indexes, lactate (Table 2), probably by expediting its conversion to butyrate and then to acetyl-CoA, which is used in the Krebs Cycle to generate ATP [31]. SA-03 supplementation was also found to reduce serum levels of another fatigue indicator, ammonia which is a waste of amino acids metabolism [32]. When starvation or exercise reduces glucose levels, skeletal muscle would metabolize amino acids to generate energy. As a result, ammonia is produced and released to blood or excreted to the urine [33]. This would cause fatigue and reduce exercise performance. A previous study showed that probiotics can decrease intestinal permeability, inhibit bacterial urease activity to reduce ammonia in blood, and increase the levels of lactic acid [34]. Since lactic acid can decrease the pH of the intestine, ammonia absorption is reduced. Probiotics have been shown to reduce inflammation and oxidative stress in liver cells, thus increased liver clearance of ammonia and other toxins [35]. Similar result as this study, SA-03 Supplementation could effectively reduce blood ammonia caused by exercise and increase glucose in the blood as energy (Figure 4A,B), and also includes two other fatigue and injury indicators, BUN and CK (Figure 5A,B), which could be significantly reduced by supplementing SA-03. Therefore, we confirm that supplementation with SA-03 can reduce fatigue and injury after exercise.

The gut microbiota and SCFA metabolites may reduce colonic mucosal permeability and inhibit inflammatory cytokines. Previous study was shown that probiotic supplementation significantly reduced serum concentrations of pro-inflammatory cytokines including, high-sensitivity CRP (hs-CRP), tumor necrosis factor α (TNF-α), interleukin (IL)-6, IL-12, and significant increase in serum levels of IL-10, as anti-inflammatory cytokine [36]. These anti-inflammatory effects may help delay fatigue symptoms in endurance performance [4]. In recent years, the functional contribution of gut-muscle axis has received more and more attention. Butyrate may have activation of multiple regulatory pathways, in addition to its anti-inflammatory properties, it can also increase ATP production and improve the metabolic efficiency of muscle fibers [37,38]. A previous study has shown that taking butyrate in aging mice has the ability to inhibit histone deacetylase, thereby improving effects such as lean muscle mass and cross-sectional area [39]. Although this difference may not be seen in this study because we used young mice (Figure 9B), this argument deserves an important reference for future studies on SA-03 with regard to sarcopenia.

Among the 26 studies we found in the literature on the effects of evaluating probiotics on exercise endurance, 17 showed no effects and nine reported significant improvements. In some studies, multiple probiotics were added to increase maximum oxygen uptake, aerobic capacity, training load, and increase physical exertion time, only a few studies point to single-strain probiotic supplementation has produced a significant aerobic performance benefit [27]. Previous studies have shown that supplementation of *Saccharomyces boulardii* in rats undergoing accelerated exercise increased maximum oxygen consumption by 12.7%, maximum aerobic speed by 12.4%, and running time to fatigue by 21.6%, compared to with sedentary the control group [40]. In our previous study, the *Bifidobacterium longum* (OLP-01) isolated from a weightlifting gold medalist found that, not only help in fatigue indicators, but also significantly increased 1.77-3.37-fold exercise to exhaustive time by 1X, 2X and 5X dose OLP-01 supplementation for four weeks, in addition, it also has a significant increase in forelimb grip, although there is no significant difference in muscle mass [16]. *Lactobacillus plantarum* (TWK10) supplement not only improves exercise performance and increases muscle mass in mice [15], but also increased endurance performance and elevated blood glucose concentration following exercise-to-exhaustion after six weeks of high dose (1 × 10^11^ CFU) TWK10 supplementation in untrained healthy male adults [41]. Four weeks of SA-03 supplementation significantly increased the time from swimming endurance to exhaustion in mice (Figure 2) and increased forelimb grip (Figure 3). Although we all know that probiotic supplementation can significantly increase SCFA and use fatty acid oxidation and activate peroxisome proliferator-activated receptor gamma coactivator-1α (PGC-1α) to generate more ATP for the energy required for exercise, and improve exercise performance and increase endurance [14,42], however, SA-03 need to be further studied as functioning auxiliary agents to enhance their functions, and may be obtained indirectly by regulating other systems.

Glycogen is a multi-branched glucose homopolysaccharide, a common form of energy storage in animals and eukaryotic microorganisms [43]. There is a high correlation between glycogen storage and carbohydrate uptake and metabolism [44]. In the intestine, carbohydrates are absorbed and converted to SCFA, such as n-butyric acid, acetic acid, and propionic acid. Propionic acid contributes to liver glycogen production [45]. As shown in Figure 6A,B, SA-03 Supplementation increased glycogen storage in liver and muscle.

At present, probiotics are used in society as a nutritional supplement. In our study, we not only explored the performance, but also observed the animal′s pathological sections and serum biochemical values at the end of the experiment. Any obvious abnormalities or obvious lesions found in various tissues will not cause any discomfort and damage to the individual. The strains selected in this research are selected from human body, and it is hoped that in the future, human experiments will be used to determine the efficacy of applying to human, which can provide exercise groups or competitive players to help improve exercise performance and capability.

## 5. Conclusions

In the present study, we found that four weeks of supplementation with SA-03 (*Lactobacillus salivarius*), a bacterial strain isolated from a weightlifting gold medalist, significantly changed the gut microbiota. Interestingly, in addition to the increased abundance of *Lactobacillus salivarius* in the SA-03 groups, the number of *Lactobacillus reuteri* also increased significantly, and to made significantly decreased the levels of fatigue indicators such as lactate, BUN, ammonia, and CK. SA-03 was also found to increase muscle strength, endurance performance, and glycogen storage in liver and muscle cells. These results suggest that SA-03 could be used as a supplement to enhance exercise performance and mitigating fatigue. Further studies are warranted to understand the mechanisms of action of SA-03.

## Figures and Tables

**Table 1:** Effect of SA-03 supplementation on various parameters.

Characteristic	Vehicle (PBS)	SA-03-1X	SA-03-2X	SA-03-5X	Trend Analysis
**Initial BW (g)**	31.7 ± 1.4	31.6 ± 1.0	31.7 ± 1.6	31.7 ± 1.3	0.8686
**Final BW (g)**	36.8 ± 2.2	36.4 ± 2.1	36.1 ± 2.3	35.9 ± 1.7	0.6070
**Water intake** (mL/mouse/day)	7.6 ± 0.5	7.3 ± 0.9	7.2 ± 0.9	7.4 ± 1.0	0.1138
**Food intake** (g/mouse/day)	8.8 ± 1.7	9.2 ± 1.2	9.0 ± 1.4	8.9 ± 1.9	0.6364
Liver (g)	1.86 ± 0.19	1.83 ± 0.22	1.89 ± 0.25	1.82 ± 0.11	0.7169
Muscle (g)	0.37 ± 0.03	0.37 ± 0.03	0.36 ± 0.04	0.37 ± 0.03	0.4901
Quadriceps (g)	0.48 ± 0.05	0.49 ± 0.05	0.47 ± 0.04	0.49 ± 0.04	0.8718
Kidney (g)	0.65 ± 0.09	0.66 ± 0.06	0.62 ± 0.03	0.62 ± 0.06	0.2541
Heart (g)	0.18 ± 0.03	0.18 ± 0.02	0.18 ± 0.02	0.18 ± 0.04	0.2773
Lung (g)	0.23 ± 0.03	0.23 ± 0.03	0.23 ± 0.03	0.23 ± 0.04	0.1584
EFP (g)	0.32 ± 0.10	0.31 ± 0.07	0.31 ± 0.07	0.31 ± 0.06	0.7057
BAT (g)	0.09 ± 0.02	0.08 ± 0.03	0.09 ± 0.02	0.09 ± 0.02	0.4066
Cecum (g)	0.85 ± 0.07	0.88 ± 0.14	0.83 ± 0.13	0.88 ± 0.10	0.8411
* Relative liver weight (%)	5.03 ± 0.25	5.04 ± 0.74	5.23 ± 0.61	5.07 ± 0.17	0.5428
Relative muscle weight (%)	1.01 ± 0.09	1.01 ± 0.04	1.01 ± 0.10	1.02 ± 0.07	0.9060
Relative quadriceps weight (%)	1.31 ± 0.14	1.34 ± 0.11	1.30 ± 0.10	1.35 ± 0.07	0.5903
Relative kidney weight (%)	1.76 ± 0.20	1.82 ± 0.11	1.73 ± 0.12	1.72 ± 0.18	0.4407
Relative heart weight (%)	0.50 ± 0.08	0.50 ± 0.04	0.51 ± 0.09	0.51 ± 0.12	0.4467
Relative lung weight (%)	0.63 ± 0.08	0.63 ± 0.08	0.63 ± 0.07	0.64 ± 0.10	0.1420
Relative EFP weight (%)	0.87 ± 0.24	0.84 ± 0.18	0.87 ± 0.23	0.86 ± 0.17	0.6941
Relative BAT weight (%)	0.25 ± 0.06	0.22 ± 0.08	0.25 ± 0.05	0.26 ± 0.06	0.3942
Relative cecum weight (%)	2.31 ± 0.19	2.43 ± 0.34	2.29 ± 0.38	2.46 ± 0.33	0.3665

Data are expressed as mean ± SD (*n* = 10 mice per group). EFP: epididymal fat pad; BAT: brown adipose tissue. * Relative to body weight.

**Table 2:** Effect of SA-03 on lactate levels.

Time Point	Vehicle	SA-03-1X	SA-03-2X	SA-03-5X	Trend Analysis
	Lactate (mmol/L)				
Before swimming (A)	3.41 ± 0.69	3.34 ± 0.56	3.30 ± 0.85	3.50 ± 0.37	0.6087
After swimming (B)	8.73 ± 0.65 ^d^	7.53 ± 0.90 ^c^	6.65 ± 0.85 ^b^	5.78 ± 0.64 ^a^	<0.0001
After 20 min resting (C)	6.84 ± 0.48 ^c^	5.85 ± 1.08 ^b^	5.40 ± 0.71 ^b^	4.53 ± 0.89 ^a^	<0.0001
**Rate of lactate production and clearance**					
Production rate = B/A	2.64 ± 0.47 ^c^	2.30 ± 0.41 ^b,c^	2.13 ± 0.57 ^b^	1.68±0.30 ^a^	<0.0001
Clearance rate = (B-C)/B	0.21 ± 0.07	0.22 ± 0.10	0.19 ± 0.08	0.22 ± 0.11	0.9901

Lactate production rate (B/A) was the value of the lactate level after exercise (B) divided by that before exercise (A). Clearance rate (B − C)/B was defined as lactate level after swimming (B) minus that after 20 min rest (C) divided by that after swimming (B). Data are expressed as mean ± SD (*n* = 10 mice per group). Values in the same row with different superscript letters (a, b, c, d) differ significantly, *p* < 0.05.

**Table 3:** Effects of SA-03 on biochemical parameters.

Parameter	Vehicle	SA-03-1X	SA-03-2X	SA-03-5X	Trend Analysis
AST (U/L)	67 ± 9	69 ± 6	67 ± 9	67 ± 9	0.7926
ALT (U/L)	36 ± 8	34 ± 9	34 ± 8	30 ± 6	0.0367
CK (U/L)	162 ± 30	165 ± 24	159 ± 16	162 ± 22	0.3890
GLU (mg/dL)	186 ± 14	186 ± 11	187 ± 14	187 ± 15	0.8841
CREA (mg/dL)	0.41 ± 0.02	0.42 ± 0.03	0.42 ± 0.02	0.42 ± 0.03	0.3146
BUN (mg/dL)	21.4 ± 3.0	21.4 ± 2.0	21.1 ± 1.9	21.3 ± 1.8	0.9156
UA (mg/dL)	1.7 ± 0.2	1.7 ± 0.2	1.7 ± 0.2	1.7 ± 0.3	0.9649
TC (mg/dL)	147 ± 22	147 ± 20	147 ± 16	147 ± 16	0.9617
TG (mg/dL)	156 ± 22	156 ± 19	156 ± 21	154 ± 21	0.7516
ALB (g/dL)	3.0 ± 0.1	3.0 ± 0.3	3.0 ± 0.3	3.0 ± 0.2	0.9723
TP (g/dL)	5.6 ± 0.3	5.7 ± 0.4	5.7 ± 0.3	5.6 ± 0.3	0.9616

Data are expressed as mean ± SD (*n* = 10 mice per group). AST, aspartate aminotransferase; ALT, alanine transaminase; CK, creatine kinase; GLU, glucose; CREA, creatinine; BUN, blood urea nitrogen; UA, uric acid; TC, total cholesterol; TG, triacylglycerol; ALB, albumin; TP, total protein.
